# Supplementary material for: Rational Design and Characterisation of Novel Mono- and Bimetallic Antibacterial Linde Type A Zeolite Materials
Source: J Funct Biomater. 2022 Jun 2;13(2):73. doi: 10.3390/jfb13020073 (PMC9224897; doi:10.3390/jfb13020073)
Supplement: Supplementary file 1 [file jfb-13-00073-s001.zip › jfb-1734457-supplementary.pdf]

SUPPORTING INFORMATION

Content :

Table S1. Complementary information on the monometallic LTA-exchanged samples prepared, concentration of the exchange solution in metal ion [M^{n+}], weight percentage of water measured by ATG, weight percentage of the metal measured by XRF and recalculated considering the water content, and molecular weight.

Table S2. Complementary information on the bimetallic LTA-exchanged samples prepared, concentration of the exchange solution in metal ion [M^{n+}], weight percentage of water measured by ATG, weight percentage of the metal measured by XRF and recalculated considering the water content, and molecular weight.

Figure S1. SEM pictures recorded for Ag-LTA-3 (1.2 wt.% Ag) and Ag-LTA-7 (11.9 wt.% Ag). EDX analyses showing Ag distribution in Ag-LTA-3 (1.2 wt.% Ag) and Ag-LTA-7 (11.9 wt.% Ag).

Figure S2. Comparison of the XRD patterns recorded for Na-LTA and Ag-LTA-7.

Table S1. Complementary informations on the monometallic LTA-exchanged samples prepared, concentration of the exchange solution in metal ion [M^{n+}], weight percentage of water measured by ATG, weight percentage of the metal measured by XRF and recalculated considering the water content, molecular weight.

name	[M^{n+}] (M)	wt.% H_2O (ATG)	wt.% M (XRF)	wt.% M (hyd)	MW (g/mol)
Na-LTA	0	20.7	0	0	2193
Ag-LTA-1	0.001	20.8	0.2	0.1	2171
Ag-LTA-2	0.005	20.5	0.8	0.6	2188
Ag-LTA-3	0.01	20.4	1.6	1.2	2200
Ag-LTA-4	0.02	20.2	3.4	2.7	2230
Ag-LTA-5	0.037	19.0	6.6	5.3	2250
Ag-LTA-6	0.05	19.6	7.7	6.2	2280
Ag-LTA-7	0.1	18.2	15.1	11.9	2352
Cu-LTA-1	0.005	20.6	0.5	0.4	2188
Cu-LTA-2	0.01	21.0	1.0	0.8	2213
Cu-LTA-3	0.025	21.1	3.2	2.5	2228
Cu-LTA-4	0.05	21.8	6.0	4.7	2249
Zn-LTA-1	0.005	20.3	0.6	0.5	2170
Zn-LTA-2	0.01	20.6	1.1	0.9	2177
Zn-LTA-3	0.025	20.0	3.1	2.5	2193
Zn-LTA-4	0.05	20.4	5.7	4.6	2226

Table S2. Complementary informations on the bimetallic LTA-exchanged samples prepared, concentration of the exchange solution in metal ion [M^{n+}], weight percentage of water measured by ATG, weight percentage of the metal measured by XRF and recalculated considering the water content, molecular weight.

name	M^{n+}	[M^{n+}] (M)	wt.% H_2O (ATG)	wt.% M (XRF)	wt.% M (hyd)	MW (g/mol)
CuAg-LTA-1	Cu	0.01	20.4	0.8	0.7	2189
	Ag	0.01		1.5	1.2	
AgCu-LTA-1	Ag	0.01	20.4	1.5	1.2	2203
	Cu	0.01		1.1	0.9	
ZnAg-LTA-1	Zn	0.01	20.0	1.1	0.9	2203
	Ag	0.01		1.9	1.5	
AgZn-LTA-1	Ag	0.01	19.9	1.8	1.4	2201
	Zn	0.01		1.3	1.1	
$Zn_{0.05}$Ag-LTA-2	Zn	0.05	20.0	4.8	3.9	2240
	Ag	0.01		1.6	1.3	
$AgZn_{0.05}$-LTA-2	Ag	0.01	19.9	1.5	1.2	2226
	Zn	0.05		4.9	3.9	

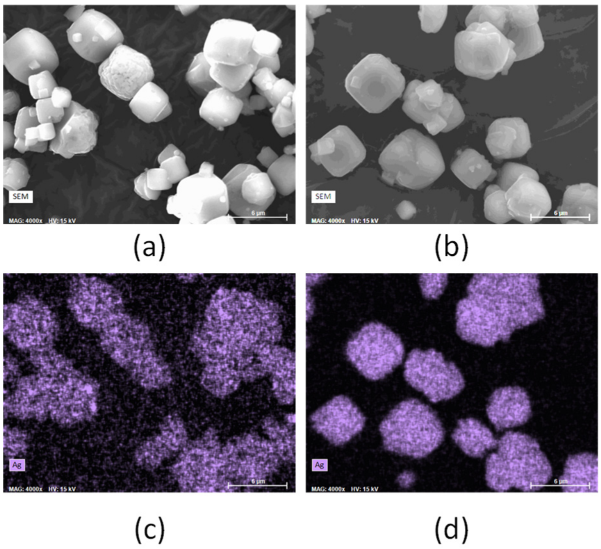

Figure S1. SEM pictures recorded for **(a)** Ag-LTA-3 (1.2 wt.% Ag) and **(b)** Ag-LTA-7 (11.9 wt.% Ag). EDX analyses showing Ag distribution in **(c)** Ag-LTA-3 (1.2 wt.% Ag) and **(d)** Ag-LTA-7 (11.9 wt.% Ag).

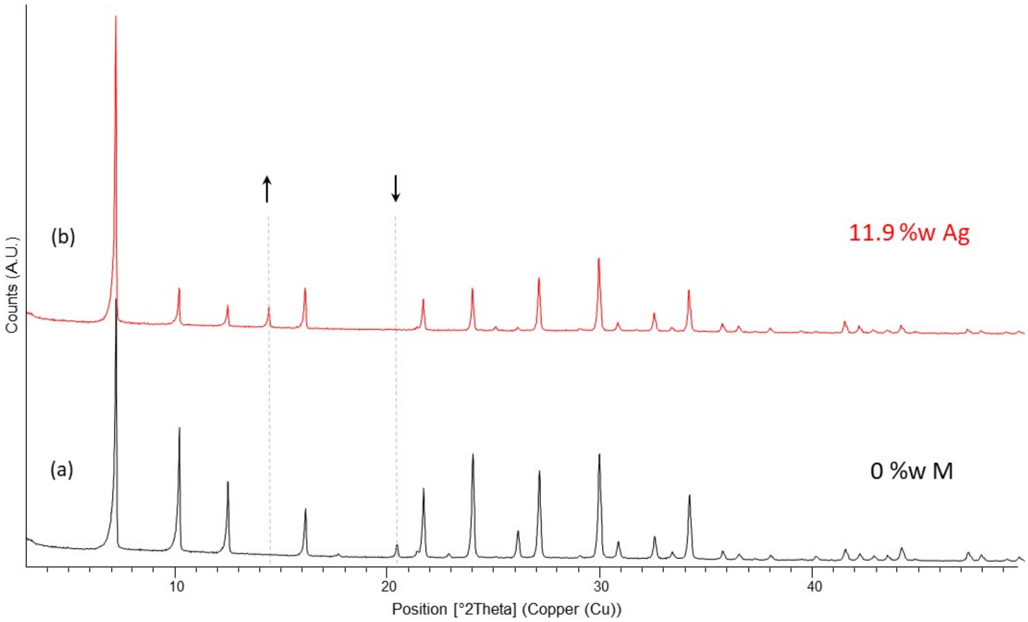

Figure S2. Comparison of the XRD patterns recorded for **(a)** Na-LTA and **(b)** Ag-LTA-7.
